# *IL-1R8* Downregulation and Concomitant *TLR7* and *TLR9* Upregulation Are Related to the Pathogenesis of Canine Diffuse Large B-Cell Lymphoma

**DOI:** 10.3390/vetsci9050209

**Published:** 2022-04-25

**Authors:** Federica Riva, Joel Filipe, Antonella Fanelli, Laura Marconato, Alessia Inglesi, Eugenio Scanziani, Sabina Soldati, Luca Licenziato, Stefano Comazzi, Lucia Minoli, Luca Aresu

**Affiliations:** 1 Department of Veterinary Medicine, University of Milan, 26900 Lodi, Italy; federica.riva@unimi.it (F.R.); joel.soares@unimi.it (J.F.); alessia.inglesi@unimi.it (A.I.); eugenio.scanziani@unimi.it (E.S.); sabina.soldati@spathology.ch (S.S.); stefano.comazzi@unimi.it (S.C.); 2 Department of Veterinary Sciences, University of Turin, 10095 Grugliasco, Italy; antonella.fanelli@unito.it (A.F.); luca.licenziato@unito.it (L.L.); luca.aresu@unito.it (L.A.); 3 Department of Medical Veterinary Sciences, University of Bologna, 40064 Ozzano dell’Emlia, Italy; laura.marconato@unibo.it; 4 Mouse and Animal Pathology Laboratory (MAPLab), Fondazione Unimi, 20133 Milan, Italy

**Keywords:** dog, diffuse large B-cell lymphoma, *IL-1R8*, *TLR7*, *TLR9*, qRT-PCR

## Abstract

Diffuse large B-cell lymphoma (DLBCL) is the most common hematological malignancy in humans and dogs. Several studies disclosed some similarities between the two species, including the constitutive activation of the NF-κB pathway as a fundamental underlying pathogenetic mechanism. In humans, the downregulation of *IL-1R8* is implicated in DLBCL development, but its role in dogs has not been explored so far. To gain insight into the pathogenesis of this tumor in dogs, we evaluated the mRNA and protein expression of IL-1R8 in 12 control lymph nodes obtained from dogs not bearing tumors and from 50 dogs with DLBCL. Moreover, we analyzed through qRT-PCR the expression of *TLR7*, *TLR9*, *MYC*, and *p52* genes that are known to be involved in the *IL-1R8* regulatory network. *IL-1R8* and *p52* were downregulated in DLBCLs compared to control lymph nodes (*p* < 0.001), while a higher expression of *TLR7*, *TLR9*, and *MYC* was observed in DLBCLs (*p* < 0.01). Immunohistochemistry confirmed the gene expression results, revealing a significantly lower IL-1R8 staining score in DLBCLs compared to control lymph nodes (*p* < 0.0001). Taken together, these results suggest that IL-1R8 downregulation may represent one of the mechanisms driving DLBCL pathogenesis in dogs, mainly through the dysregulation of the Toll-like/interleukin receptors signaling cascade and the aberrant activation of the classical NF-κB pathway.

## 1. Introduction

Diffuse large B-cell lymphoma (DLBCL) is the most frequent non-Hodgkin lymphoma subtype worldwide, accounting for approximately 40% of all human cases [1]. The name, DLBCL, stems from the presence of large neoplastic B cells, which are two or three times larger than red blood cells, and the diffuse pattern, destroying nodal and, in some cases, extra nodal tissues [1]. Similarly, DLBCL is the most common canine aggressive lymphoproliferative tumor and is characterized by a heterogeneous clinical course that cannot be deciphered by morphology and immunophenotype alone [2,3,4,5]. The disease is aggressive, and, although some dogs achieve long-term remission, the majority succumb to DLBCL [6].

In human DLBCL, the neoplastic transformation of B cells is mainly driven by genetic modifications and epigenetic reprogramming [7]. Analogies between human and canine DLBCL (cDLBCL) have been identified, and the main distinctive feature in both species emerged from tumor transcriptome studies, where the constitutive activation of the classical NF-κB pathway was demonstrated as a common hallmark [8]. After the engagement of cell surface receptors, a cascade of events, including the activation of the IKK complex, IkBa phosphorylation, ubiquitination, and proteasomal degradation, allows NF-κB dimers to translocate to the nucleus, initiating the transcription of several genes [8]. The causes of the aberrant NF-κB activity have not yet been completely resolved in dogs, but canonical NF-κB signaling resulted in enhanced cDLBCL compared to the non-canonical pathway, and both cell-extrinsic and cell-intrinsic stimuli were supposed to contribute [9]. Therefore, the elucidation of the mechanisms underlying NF-κB activation may result in a high clinical relevance, possibly identifying specific therapeutic targets.

IL-1R8, also known as SIGIRR or TIR8, belongs to the IL-1 receptor superfamily [10] and is highly conserved among vertebrates [11]. Physiologically, IL-1R8 inhibits NF-κB activation and other transcription factors that are induced by Toll-like/interleukin-1 receptor (TIR) signaling through different mechanisms, such as interfering with the TLR/ILR extracellular domain dimerization or blocking the recruitment of TIR-domain-containing adaptor molecules, eventually blocking TLR and ILR signaling cascades [12,13,14,15]. Conversely, IL-1R8 deficiency has been associated with several pathological conditions in mice, including infections, autoimmune diseases, sterile inflammations, and cancers. In particular, IL-1R8 deficiency has been associated with B-cell lymphoma development and tumor progression in murine models of chronic lymphocytic leukemia [16].

To date, the role of IL-1R8 in cDLBCL has never been investigated, but we recently hypothesized the induction of proliferation in canine nodal B-cell lymphomas upon the triggering of several TLRs [17]. Given the ability of IL-1R8 to tune the activation of TLRs, we sought to gain insight into the potential role of this molecule in the pathogenesis of cDLBCL through an integrated analysis of mRNA and protein expression, complemented by a comparison with normal canine lymph nodes. Moreover, genes known to be involved in the regulatory mechanisms of IL-1R8 and cDLBCL pathogenesis, including *TLR7*, *TLR9*, *MYC*, and *p52* were investigated at the mRNA level. Finally, potential associations with clinico-pathological features were analyzed.

## 2. Materials and Methods

Fifty dogs with newly diagnosed and previously untreated multicentric DLBCL were included in the study. A DLBCL diagnosis was based on morphology and immunophenotype, according to the World Health Organization (WHO) classification criteria for canine lymphoma [18]. Dogs with DLBCL of any WHO clinical stage were enrolled [19]. These dogs underwent a complete staging work-up (history and physical examination, complete blood cell count, serum biochemistry profile, thoracic radiographs and abdominal ultrasound, cytological evaluation of the liver and spleen, and immunophenotype determined by flow cytometry on lymph node aspirate, peripheral blood, and bone marrow aspirate). An enlarged lymph node (LN) was always obtained from each dog for diagnostic purposes and further analyses. Twelve control LNs were obtained from dogs not bearing tumors that died of unrelated causes and were undergoing necropsy. Grossly, the control lymph nodes were within the normal limits, and a light microscopy examination excluded lesions other than mild reactive hyperplasia. Tissue samples from the cDLBCL and control dogs were divided in two portions: one was fixed in formalin for histological and immunohistochemical analyses, and one was stored in RNAlater (Sigma-Aldrich, St. Louis, MO, USA) at −80 °C for RNA extraction [20].

The dogs were treated either with chemotherapy (n = 20) or with chemo-immunotherapy (n = 30), depending on the owner’s choice. Unvaccinated dogs received a CHOP-based protocol, including L-asparaginase, vincristine, cyclophosphamide, doxorubicin, lomustine, and prednisone. Chemo-immunotherapy consisted of a CHOP-based protocol with the addition of an APAVAC vaccine, as previously described [21]. At the end of treatment, all dogs underwent end-staging, including flow cytometry (FC) on a peripheral LN, peripheral blood, and bone marrow and imaging. The follow-up evaluation consisted of a monthly physical examination, peripheral LN size measurement, and cytological evaluation during the first year and every other month thereafter. Relapse was defined as the clinical reappearance and cytological evidence of lymphoma with or without FC confirmation in any anatomical site in dogs that experienced complete remission. The time to progression (TTP) was calculated as the interval between the initiation of treatment and progressive disease or relapse, whereas lymphoma-specific survival (LSS) was measured as the interval between the initiation of treatment and lymphoma-related death.

This study complied with Italian laws on animal experimentation and ethics and was approved by the Ethical Committee of the Università degli Studi di Milano (OPBA_61_2018 25 June 2018).

### 2.1. RNA Extraction and Reverse Transcription

Control nodes were processed using the guanidine isothiocyanate method. Briefly, the samples were homogenized in 5 mL of guanidine isothiocyanate (4 M, pH 7) (Sigma-Aldrich, St. Louis, MO, USA) using a rotor-stator system (Ultra Turrax, IKA WERKE, Staufen, Germany). Then, 1.3 mL of lysate was centrifuged for 16 h at 42,000 rpm at 18 °C upon a 1 mL 5.7 M cesium chloride layer (Invitrogen, Paisley, Scotland, UK) by ultracentrifugation (Beckman Instruments, Inc., Palo Alto, CA, USA). The supernatant was discarded, and the RNA pellet was dissolved in sterile water and precipitated with absolute ethanol (Carlo Erba S.p.a., Chaussée du Vexin, France) and sodium acetate (3 M, pH 5.4) (Carlo Erba S.p.a., Chaussée du Vexin, France) in dry ice for 2 h. After centrifugation at 12,000× *g* at 4 °C for 30 min, the RNA pellet was washed in 1 mL of 70% ethanol and dissolved in sterile water.

The cDLBCL tissues were processed using the TRIzol (Sigma-Aldrich, St. Louis, MO, USA) method following the manufacturer’s instructions. The RNA concentration was established by measuring absorbance at 260 nm with a spectrophotometer (BioPhotometer, Eppendorf, Hamburg, Germany), while the RNA integrity was assessed through 2% agarose gel electrophoresis (GellyPhor LM Agarose, Euroclone S.p.a., Pero-Milano, Italy). The samples were stored at −20 °C until use. Then, 2 μg of total RNA from each sample was reverse transcribed using the High-Capacity cDNA Reverse Transcription Kit (Applied Biosystem, Foster City, CA, USA) according to the manufacturer’s instructions using random primers. The reverse transcription was performed according to the following program: step 1, incubation at 25 °C for 10 min; step 2 at 37 °C for 2 h; and step 3 at 85 °C for 5 min.

### 2.2. Real-Time qRT-PCR

The cDNA obtained from each sample was used as a template for real-time PCR in an optimized 25 μL reaction volume using Sybr Green chemicals (ThermoFisher Scientific, Waltham, MA, USA), as previously described [22]. Primer pairs for canine *p52*, *MYC*, *IL-1R8*, *TLR7*, *TLR9* and the housekeeping *CCZ1* genes were designed using the Primer Express Software (Applied Biosystem, Foster City, CA, USA) and were purchased from Invitrogen (Carlsbad, CA, USA). The primer sequences are listed in Table S1. Quantitative RT-PCR was performed using the ABIPRISM 7000 Sequence Detection System (Applied Biosystems, Foster City, CA, USA). A duplicate no-template control (NTC) was included in each experiment. The analysis of the gene expression data was performed using the 2^−ΔCt^ method (Livak method [23]). Briefly, ΔCt was calculated for each gene for each sample (cDLBCLs and control lymph nodes) using the *CCZ1* housekeeping gene for normalization. The mean 2^−ΔCt^ ± SD was calculated for each gene in the cDLBCL and control lymph nodes groups.

### 2.3. Immunohistochemistry

The cDLBCL and control lymph node samples were formalin-fixed and paraffin embedded for histopathologic examination and immunohistochemistry to evaluate IL-1R8 expression. Deparaffinization, rehydration, and antigen retrieval were performed in a dewaxing buffer solution (pH = 9) (Dewax and HIER Buffer H, Thermo Fisher Scientific, Cheshire, UK) at 100 °C for 40 min. The samples were cooled and subsequently washed in PBS and 0.005% Tween20 (Agilent, Santa Clara, CA, USA). The sections were then stained with the Thermo Scientific Autostainer 480S system (Bio Optica, Milan, Italy). After peroxidase inhibition and non-specific binding blocking, the sections were incubated with the primary anti-SIGIRR antibody (Polyclonal Rabbit AHP1784-BioRad, Hercules, CA, USA; dilution 1:900) for 1 h at room temperature. The samples were then incubated with a goat anti-rabbit biotinylated secondary antibody (Vector Lab BA-2000, Vector Laboratories, Burlingame, CA, USA; dilution 1:200) and with an avidin-biotin peroxidase system (Vectastain^®^ Elite^®^ ABC Kit, Vector Laboratories. Burlingame, CA, USA). The immunolabelling was revealed by incubation with 3,3-diaminobenzidine tetra-hydrochloride (DAB; Vector Laboratories) for 3 min. After washing in distilled water, the sections were counterstained with Mayer’s hematoxylin (Diapath S.p.A, Martinengo, Italy), dehydrated in a graded alcohol series, and mounted. Normal lymph nodes of known immunoreactivity were included as positive controls in each immunohistochemical run. The stained tissue sections were analyzed at the optical microscope (Leica, Wetzlar, Germany). A semiquantitative evaluation of IL-1R8 cytoplasmic expression was performed: the final score was obtained as the product of the number of positive cells (score 0 < 1%; 1 = 1–10%; 2 = 11–50%; 3 > 50%) and the intensity of the staining (score 0 = absent; 1 = slight; 2 = moderate; 3 = strong).

### 2.4. Statistical Analysis

Statistical analyses were performed in the R environment. The continuous variables were tested for normal distribution by conducting a Shapiro–Wilk test. The gene expression results of the cDLBCL and control lymph nodes were compared by means of a Mann–Whitney test. The correlation matrix between the target genes was computed using the *ggcorrplot* R package, and significant associations were checked using a Spearman correlation test. A Benjamini–Hochberg correction was applied to multiple testing. To explore the associations between gene expression and clinico-pathological variables (breed, age, sex, weight, stage, substage, bone marrow and peripheral blood infiltration, serum LDH levels, pretreatment with steroids, and treatment), a Mann–Whitney test was performed for the categorical variables, with the exception of ‘stage’ where a Kruskall–Wallis test was used, while a Spearman correlation test was conducted for the continuous variables. Finally, the impact of the clinico-pathological variables and gene expression levels on both TTP and LSS was explored by means of univariate and multivariate Cox proportional-hazards models. Dogs that were lost to follow-up or died of lymphoma-unrelated causes before progressive disease as well as those still in complete remission at the end of the study were censored for the TTP analysis. Dogs that were alive at the end of the study, lost to follow-up, or dead due to causes other than lymphoma were censored for the LSS analysis [24]. Differences in the immunohistochemical expression of IL-1R8 were evaluated by a Mann–Whitney test.

## 3. Results

### 3.1. Study Population

Fifty dogs with newly diagnosed, previously untreated DLBCL were enrolled. Detailed signalment and clinico-pathological data are reported in Table S2. Mixed-breed dogs (n = 11, 22%), German shepherd dogs (n = 7, 14%), Rottweilers (n = 4, 8%) and Golden Retrievers (n = 4, 8%) were the most represented breeds. In total, 26 dogs were males (52%), while 24 were females (48%). The median age was 7.5 years (range: 3–15 years), while the median weight was 28.9 kg (range: 4.5–81.3 kg). It was found that 32 dogs (64%) had stage V disease, 16 dogs (32%) had stage IV, and 2 dogs (4%) had stage III disease. In addition, 33 dogs (66%) were asymptomatic, while 17 (34%) presented clinical symptoms. Fifteen dogs (30%) presented bone marrow infiltration (i.e., >3% neoplastic cells) (median: 1.4%; range: 0.2–50%) and peripheral blood infiltration (median: 1.0%; range: 0.1–55.5%). Twenty-six dogs (52%) showed an increased level of serum LDH. Finally, 14 dogs (28%) had received steroids before lymphoma diagnosis. The median TTP was 171 days, while the median LSS was 237 days. The TTP was significantly affected by treatment (*p* = 0.02) (Table S3a), while the LSS was significantly affected by the percentage of bone marrow infiltration (*p* = 0.03) (Table S3b).

### 3.2. Gene Expression Analysis

**Figure 1:**
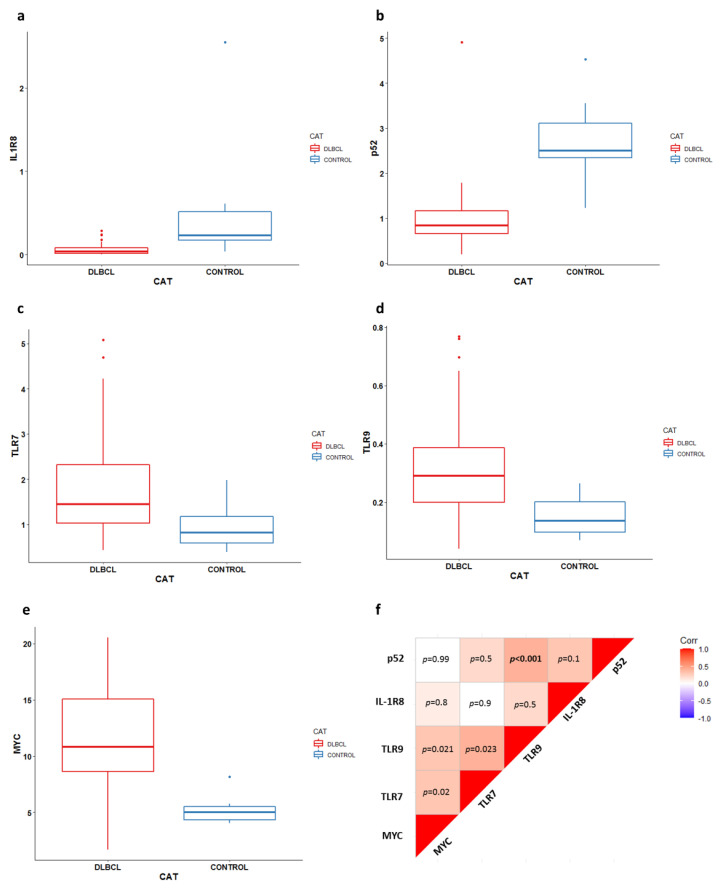
Gene expression results and correlations between target genes. (**a**–**e**) Real-time PCR results of *IL-1R8* (**a**), *p52* (**b**), *TLR7* (**c**), *TLR9* (**d**), and *MYC* (**e**) in 50 cDLBCLs and 12 control lymph nodes; CAT = category. (**f**) Correlation matrix showing statistical correlations between target gene expressions. Correlation coefficients were calculated using the Spearman method.

We evaluated the expression of the *IL-1R8*, *TLR7*, *MYC*, *TLR9*, and *p52* genes in cDLBCL and control lymph nodes through qRT-PCR. The gene expression results are reported in Figure 1a–e and Table S4. *IL-1R8* and *p52* were significantly downregulated in tumors compared to control lymph nodes (*p* < 0.001). Conversely, *TLR7*, *TLR9*, and *MYC* showed higher expressions in cDLBCL compared to controls (*p* < 0.01). Interestingly, a significant correlation between *TLR9* and *p52* (rho = 0.49, *p* < 0.001) was retrieved (Figure 1f and Table S5). Conversely, no association between gene expression and any clinico-pathological variable was obtained. Likewise, none of the target genes significantly affected the outcome (Table S3a,b).

### 3.3. Immunohistochemistry

**Figure 2:**
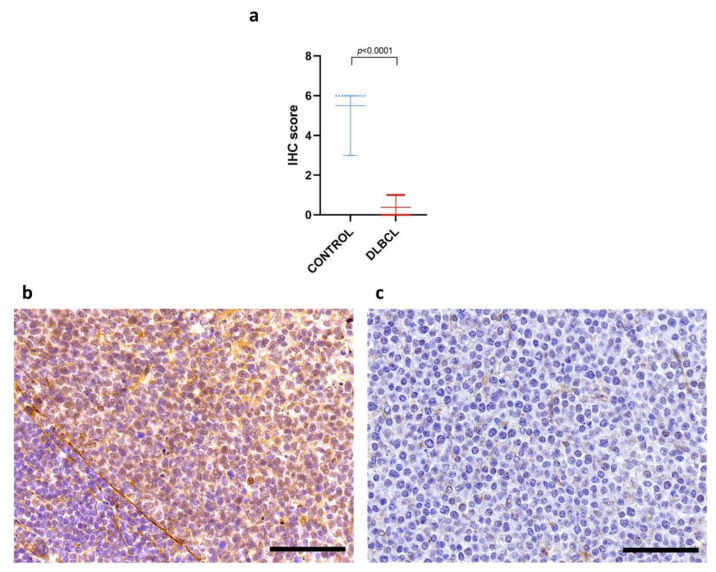
Immunohistochemical expression of IL-1R8 in DLBCLs and control lymph nodes. (**a**) Scatter plot of immunohistochemical semiquantitative score in control lymph nodes and canine DLBCL. Data are shown as individual values with mean and range. (**b**) Diffuse expression of IL-1R8 in germinal center B cells in a control lymph node. Immunohistochemistry for IL-1R8, 400× magnification. Bar = 200 µm. (**c**) Lack of IL-1R8 expression in neoplastic lymphocytes in cDLBCL. Immunohistochemistry for IL-1R8, 400× magnification. Bar = 200 µm.

In line with the gene expression results, the immunohistochemical analysis confirmed a significant down regulation of IL-1R8 protein in cDLBCL compared to control lymph nodes (*p* < 0.0001) (Figure 2a). In the latter, B cells within the germinal centers were diffusely and moderately positive for IL-1R8, while only scattered positive lymphocytes were detected in the remaining regions of the lymph node, including the paracortex (Figure 2b). Therefore, the final score was obtained on selected areas that were compatible with lymphoid follicles and ranged from 3 to 6 (mean = 5.5). The immunohistochemical score ranged from 0 to 1 (mean = 0.38) in cDLBCL (Figure 2c).

## 4. Discussion

Persistent activation of the NF-κB signaling pathway has been identified as a fundamental molecular event that is required to amplify the survival and proliferation of neoplastic cells in both humans and dogs with DLBCL [25]. However, the mechanisms driving aberrant activation are scarcely investigated in dogs, and two hypotheses have been proposed so far. The first correlates the presence of *TRAF3*-inactivating mutations with NIK stabilization and constitutive NF-κB activity via the alternative pathway [26]. The second hypothesis is more intriguing and associates the activation of NF-κB with the enrichment of the TLR signaling pathway in neoplastic B cells. Indeed, several TLRs were found to be upregulated in dogs with DLBCL, and, consequently, a pathogenesis mimicking a chronic antigen-like stimulation was supposed [17].

Here, to gain further insights into the mechanisms involved in NF-κB activation in cDLBCL, we first investigated one of the most well-known negative TLR regulators, named IL-1R8, at both the gene and protein levels. As hypothesized, *IL-1R8* mRNA expression was significantly downregulated in tumors compared to control lymph nodes. Likewise, at the protein level, IL-1R8 immunostaining was prevalently detected in normal B cells within the germinal centers. In contrast, in cDLBCLs the IL-1R8 signal varied from absent to very low. In particular, both the staining intensity and number of positive cells were significantly lower compared with normal B cells. These results are in line with previous studies in human tumors, where a reduced *IL-1R8* expression was reported in DLBCLs when compared to peripheral blood mononuclear cells and germinal center B cells and correlated with a better overall survival, suggesting its role in the pathogenesis of this lymphoma subtype [27]. In addition, a deficiency in IL-1R8 predisposed *Fas^lpr^* mice to severe lymphoproliferation and autoimmune lupus-like disease and TCL1 mice to leukemia [14,16,27].

In our experiments, we obtained similar results, but we did not investigate the mechanisms causing *IL-1R8* silencing. However, we can almost exclude methylation aberrancies and genetic mutations as possible driving events. Indeed, the methylome of these tumors was previously investigated by methyl binding protein sequencing, but no peaks in the promoter regions were identified in the *IL-1R8* locus [17]. Moreover, whole exome studies did not report genetic mutations affecting this gene in canine B-cell lymphoma [26,28,29]. One last hypothesis refers to genetic or epigenetic alterations in specific genes that interact directly with *IL-1R8* and favor its gene transcription, such as *SP1*. The role of SP1 in the regulation of *IL-1R8* was recently confirmed in human primary monocytes and neutrophils, and its pharmacological inhibition was able to reduce *IL-1R8* expression [30]. No data are available about the regulation of this transcription factor in veterinary medicine, and future experiments are required.

Since IL-1R8 physiologically inhibits several TLRs [10,12], we investigated here if this was also true in dogs. The inverse correlation between TLRs and IL-1R8 indirectly confirmed the same constitutive mechanisms described in humans and mice, where IL-1R8 negatively modulates the activation of TLR7 and TLR9, in turn leading to the inhibition of NF-κB and JNK signaling [14,31]. Therefore, we can speculate that the reduction of IL-1R8 may participate in the mechanisms beyond TLR upregulation in cDLBCL. Furthermore, significantly higher *TLR7* and *TLR9* mRNA levels were observed in tumors compared to controls, confirming that their activity is not only restricted to normal immune system cells but is also identified in tumors [17]. In this respect, these data provide evidence of clinical relevance, which will be useful in devising therapeutic trials with TLR9 agonists, such as oligodeoxynucleotides containing CpG motifs (CpG-ODN), in dogs with DLBCL. Hypothesizing a similar mechanism to human B-cell lymphomas, we can suppose an inhibition of proliferation and induction of apoptosis in TLR9-positive neoplastic cells after treatment.

To further confirm the activation of the NF-κB classical pathway in cDLBCL, we tested *p52* mRNA expression. The NF-κB-subunits involved in the canonical NF-κB pathway are principally RelA, p65, and p105/p50, whereas p52 and p100 are always undetectable. Overall, the results showed a downregulation of *p52* in cDLBCL compared to controls, confirming the NF-κB canonical pathway activation.

In order to test other genes that are known to be involved in DLBCL pathogenesis, we analyzed *MYC* mRNA levels in the same samples [32]. Our results confirmed the upregulation of this proto-oncogene in tumors, as previously described by array comparative genomic hybridization and whole exome sequencing studies, where amplifications of chromosome 13 or specific mutations were catalogued [17,26,28,32]. The opposite correlation between *MYC* and *IL-1R8* in cDLBCLs replicate the results obtained in human colon cells where increased *MYC* levels driven by the ERK/MAPK signaling pathway were reported in IL-1R8-deficient Apc^Min/+^colonocytes [33]. More specifically, IL-1R8 deficiency is able to stimulate the activation of ERK that, in turn, stabilizes MYC, thereby preventing its degradation. We could not prove it here, but ERK aberrant activity is fundamental in the pathogenesis of canine B-cell lymphoma, as previously demonstrated by Assumpção et al. [34].

In conclusion, similar to humans, *IL-1R8* has a reduced expression in cDLBCL. The results obtained here reinforce the previously formulated hypothesis in dogs with DLBCL, suggesting that *TLR7* and *TLR9* upregulation and the concomitant *p52* and *IL-1R8* downregulation in B cells could lead to an exaggerated and uncontrolled inflammatory-like process that is characterized by activated B cells, eventually evolving into cancer. The tumorigenesis might be further amplified by *MYC* deregulation. Further experiments are needed to clarify the mechanisms causing *IL-1R8* downregulation and stimuli activating TLRs, including a possible aberrant activation driven by a virus integrated in the tumor genome.

## Data Availability

Not applicable.

## Supplementary Materials

The following supporting information can be downloaded at: https://www.mdpi.com/article/10.3390/vetsci9050209/s1, Table S1: Primer pair list; Table S2: Signalment and clinico-pathological variables of 50 dogs with DLBCL included in the study; Table S3: Median TTP and LSS and survival analysis of 50 dogs with cDLBCL; Table S4: *IL-1R8*, *p52*, *TLR7*, *TLR9*, and *MYC* expression in cDLBCLs and control lymph nodes; Table S5: Spearman correlation results of *IL-1R8*, *p52*, *TLR7*, *TLR9*, and *MYC* expression in cDLBCLs.
