# Supplementary material for: IL-1R8 Downregulation and Concomitant TLR7 and TLR9 Upregulation Are Related to the Pathogenesis of Canine Diffuse Large B-Cell Lymphoma
Source: Vet Sci. 2022 Apr 25;9(5):209. doi: 10.3390/vetsci9050209 (PMC9147662; doi:10.3390/vetsci9050209)
Supplement: Supplementary file 1 [file vetsci-09-00209-s001.zip › Table S1.pdf]

Table S1. Primer pair list.

Gene	***Protein***	***Sequence (5' → 3')***	**Gene ID**	***Amplicon***
TLR7	Toll-like receptor 7	F:TGGAAATTGCCCTCGTT<br>R:GTCAATGCATCGAAAGCTGA	491743	*99 bp*
TLR9	Toll-like receptor 9 protein	F:GCAATACCCCGAGCCTGATG<br>R:TATGCAGGCGATTCTGGGAC	403502	*105 bp*
C-MYC	myc proto-oncogene protein	F:GCGACTCGGAGGAAGAACAA<br>R:GACCCCGATTCGGACCTTTT	403924	*94 bp*
NFKB2	nuclear factor NF-kappa-B p100 subunit	F:GCCCCTGAAGCCAGTTATTTC<br>R:CCAGGAGACTTGCTGTCATGG	486858	*51 bp*
IL-1R8	Interleukine-1 receptor 8	F:GATGACAAGGACCCCATGCT<br>R:TTGCATGCGGTGGAGCTAAT	100686933	*142 bp*
CCZ1	CCZ1 Homolog, Vacuolar Protein Trafficking And Biogenesis Associated	F:TGAAGGCACTGCATTTAATTGTTTAT<br>R:CTTCGGCAAAAATCCAATGT	479750	*76 bp*
