# Supplementary material for: IL-1R8 Downregulation and Concomitant TLR7 and TLR9 Upregulation Are Related to the Pathogenesis of Canine Diffuse Large B-Cell Lymphoma
Source: Vet Sci. 2022 Apr 25;9(5):209. doi: 10.3390/vetsci9050209 (PMC9147662; doi:10.3390/vetsci9050209)
Supplement: Supplementary file 1 [file vetsci-09-00209-s001.zip › Table S4.pdf]

Table S4. *IL-1R8, p52, TLR7, TLR9* and *MYC* expression in cDLBCLs and control lymph nodes. Data are expressed as relative quantification values (arbitrary units, mean ± SD).

	Mean ± SD			
	**DLBCLs**	**Control LNs**	**Fold change**	**p-value**
IL-1R8	0.06 ± 0.07	0.48 ± 0.68	-7.47	p=1.123E-05
p52	1.00 ± 0.68	2.70 ± 0.83	-2.69	p=7.638E-07
TLR7	1.78 ± 1.09	0.93 ± 0.47	1.92	p=0.004485
MYC	11.61 ± 4.62	5.15 ± 1.13	2.26	p=5.772E-06
TLR9	0.32 ± 0.17	0.15 ± 0.07	2.16	p=0.0002687
