# Supplementary material for: IL-1R8 Downregulation and Concomitant TLR7 and TLR9 Upregulation Are Related to the Pathogenesis of Canine Diffuse Large B-Cell Lymphoma
Source: Vet Sci. 2022 Apr 25;9(5):209. doi: 10.3390/vetsci9050209 (PMC9147662; doi:10.3390/vetsci9050209)
Supplement: Supplementary file 1 [file vetsci-09-00209-s001.zip › Table S5.pdf]

**Table S5. Spearman correlation results of *IL-1R8, p52, TLR7, TLR9* and *MYC* expression in cDLBCLs. Significant correlations after Benjamini-Hochberg correction are highlighted in bold.**

| | | **rho** | **p-value** |
|---|---|---|---|
| ***TLR7*** | ***MYC*** | 0.329 | 0.02 |
| ***TLR7*** | ***TLR9*** | 0.32 | 0.023 |
| ***TLR7*** | ***IL1R8*** | -0.019 | 0.89 |
| ***TLR7*** | ***p52*** | 0.088 | 0.54 |
| ***MYC*** | ***TLR9*** | 0.325 | 0.021 |
| ***MYC*** | ***IL1R8*** | -0.041 | 0.78 |
| ***MYC*** | ***p52*** | 0.002 | 0.99 |
| ***TLR9*** | ***IL1R8*** | 0.106 | 0.46 |
| ***TLR9*** | ***p52*** | 0.495 | **0.00026** |
| ***IL1R8*** | ***p52*** | 0.222 | 0.12 |
